# Reliability of structural MRI measurements: The effects of scan session, head tilt, inter-scan interval, acquisition sequence, FreeSurfer version and processing stream

**DOI:** 10.1016/j.neuroimage.2021.118751

**Published:** 2022-02-01

**Authors:** Emily P Hedges, Mihail Dimitrov, Uzma Zahid, Barbara Brito Vega, Shuqing Si, Hannah Dickson, Philip McGuire, Steven Williams, Gareth J Barker, Matthew J Kempton

**Affiliations:** a Department of Psychosis Studies, Institute of Psychiatry, Psychology & Neuroscience, King's College London, 16 De Crespigny Park, London SE5 8AF, United Kingdom; b Department of Forensic and Neurodevelopmental Sciences, Institute of Psychiatry, Psychology & Neuroscience, King's College London, London SE5 8AF, United Kingdom; c Department of Neuroimaging, Centre for Neuroimaging Sciences, Institute of Psychiatry, Psychology & Neuroscience, King's College London, London SE5 8AF, United Kingdom

**Keywords:** Structural MRI, Longitudinal, Reproducibility, Morphology

## Abstract

**Background:**

Large-scale longitudinal and multi-centre studies are used to explore neuroimaging markers of normal ageing, and neurodegenerative and mental health disorders. Longitudinal changes in brain structure are typically small, therefore the reliability of automated techniques is crucial. Determining the effects of different factors on reliability allows investigators to control those adversely affecting reliability, calculate statistical power, or even avoid particular brain measures with low reliability. This study examined the impact of several image acquisition and processing factors and documented the test-retest reliability of structural MRI measurements.

**Methods:**

In Phase I, 20 healthy adults (11 females; aged 20–30 years) were scanned on two occasions three weeks apart on the same scanner using the ADNI-3 protocol. On each occasion, individuals were scanned twice (repetition), after re-entering the scanner (reposition) and after tilting their head forward. At one year follow-up, nine returning individuals and 11 new volunteers were recruited for Phase II (11 females; aged 22–31 years). Scans were acquired on two different scanners using the ADNI-2 and ADNI-3 protocols. Structural images were processed using FreeSurfer (v5.3.0, 6.0.0 and 7.1.0) to provide subcortical and cortical volume, cortical surface area and thickness measurements. Intra-class correlation coefficients (ICC) were calculated to estimate test-retest reliability. We examined the effect of repetition, reposition, head tilt, time between scans, MRI sequence and scanner on reliability of structural brain measurements. Mean percentage differences were also calculated in supplementary analyses.

**Results:**

Using the FreeSurfer v7.1.0 longitudinal pipeline, we observed high reliability for subcortical and cortical volumes, and cortical surface areas at repetition, reposition, three weeks and one year (mean ICCs>0.97). Cortical thickness reliability was lower (mean ICCs>0.82). Head tilt had the greatest adverse impact on ICC estimates, for example reducing mean right cortical thickness to ICC=0.74. In contrast, changes in ADNI sequence or MRI scanner had a minimal effect. We observed an increase in reliability for updated FreeSurfer versions, with the longitudinal pipeline consistently having a higher reliability than the cross-sectional pipeline.

**Discussion:**

Longitudinal studies should monitor or control head tilt to maximise reliability. We provided the ICC estimates and mean percentage differences for all FreeSurfer brain regions, which may inform power analyses for clinical studies and have implications for the design of future longitudinal studies.

## 1 Introduction

Longitudinal and multi-centre studies have become increasingly popular to explore neuroimaging markers of normal ageing, and neurodegenerative and mental health disorders (Misra et al., 2009; Scahill et al., 2003; van Erp et al., 2016). Due to the large number of participants in such studies, quantifying brain structures by time-consuming manual tracing methods becomes unfeasible (Morey et al., 2009). Instead, automated brain segmentation techniques are used to process magnetic resonance imaging (MRI) scans (Jovicich et al., 2013; Morey et al., 2010). FreeSurfer is an open-source automated segmentation software package (Martinos Center for Biomedical Imaging, Harvard-MIT, Boston; https://surfer.nmr.mgh.harvard.edu/) that has been widely used in brain volume studies, such as UK Biobank (Elliott et al., 2018), ENIGMA Consortium (Thompson et al., 2014) and Lifebrain (Walhovd et al., 2018). FreeSurfer is appealing to longitudinal and multi-centre studies as it offers a fully automated longitudinal processing stream for structural MRI images (Reuter et al., 2012) that extracts reliable brain morphometry estimates (Jovicich et al., 2013; McCarthy et al., 2015). In addition, by using the same template system, FreeSurfer provides consistent results as it is not subject to inter-rater variability, unlike manual tracing (Lehmann et al., 2010; Morey et al., 2009). Nevertheless, combining data in longitudinal studies may contribute additional sources of variance, such as changes in MRI sequence, that could limit the reliability of brain measurements. Therefore, it is important for clinical neuropsychiatric research to determine factors that may affect the reliability of FreeSurfer-derived measurements so we can increase the sensitivity of longitudinal MRI to real disease-related changes (Jovicich et al., 2006).

Previous reliability studies of structural MRI have demonstrated that changes in image acquisition variables, such as scan session, MRI sequence (Jovicich et al., 2009) and head motion during scanning (Alexander‐Bloch et al., 2016; Reuter et al., 2015) may affect brain measurement reliability. Moreover, changes in FreeSurfer segmentation methods, such as inter-version variation (Chepkoech et al., 2016; Whelan et al., 2016) and processing stream (Jovicich et al., 2013), may also affect the reproducibility of brain measurements. To assess the reliability of repeated measures, intra-class correlation coefficients (ICC) (McGraw and Wong, 1996) are a favourable metric that have been widely used in research from genetics (Gibert et al., 1998) to clinical rating scales (Nuechterlein et al., 2008), as well as in neuroimaging studies (Du et al., 2020; Morey et al., 2010; Sederevičius et al., 2021; Takao et al., 2021). However, reliability studies to date are significantly limited: studies have focused on older versions of FreeSurfer software and commonly report on only a few regional volume or thickness estimates. Yet, it is imperative to quantify and report the reliability of all segmented brain regions, as well as examining the effects of a range of image acquisition and processing factors on reliability. This will allow future studies to address factors that may reduce the reliability of MRI-derived measurements and so increase sensitivity of longitudinal MRI to real changes of interest. In addition, ICC reliability values may be used to inform power analyses to ensure clinical studies have sufficient statistical power to detect small group differences (Liem et al., 2015).

The purpose of the present study was to examine the effects of image acquisition variables (i.e., scan session, subject positioning and head tilt, MRI sequence and scanner) and FreeSurfer processing variables (i.e., software version and processing stream) on brain morphometry estimates. For the Precision in Neuroimaging study, we adopted a prospective, longitudinal design where healthy participants were scanned at baseline, three weeks and one year. For MRI acquisition, we chose to use the third generation of the Alzheimer's Disease Neuroimaging Iniative (ADNI) protocol (http://adni.loni.usc.edu/) as the ADNI-3 sequence was specifically developed for longitudinal multi-centre studies (Jack Jr et al., 2008). However, for comparison, we also collected MRI data using the second generation of the ADNI protocol as the ADNI-2 sequence has already been used in the ADNI consortium and several international studies of mental health disorders [e.g., EU-GEI High Risk Study (Modinos et al., 2020) and PSYSCAN (Tognin et al., 2020)]. Processing of structural MRI data was independently carried out for the cross-sectional and longitudinal streams of three FreeSurfer versions (v5.3.0, v6.0.0, v7.1.0). We have conducted a comprehensive assessment of reliability for all FreeSurfer-segmented cortical and subcortical volumes, cortical surface area, and cortical thickness estimates. Results were derived from ICC estimates of individual brain regions that are freely available in a supplement to this paper. Furthermore, the defaced neuroimaging data for a subset of participants who provided consent has been made publicly available for researchers to download (https://sites.google.com/view/pinstudy).

## 2 Methods

### 2.1 Participants

The Precision in Neuroimaging (PIN) study consisted of two phases. Participant recruitment for Phase 1 began in December 2017 and Phase II in March 2019. At Phase I, twenty participants were recruited from King's College London and from the general public. At Phase II, nine Phase I participants were successfully re-recruited. Phase I participants who did not wish to take part in Phase II were replaced with new participants.

For Phase I and Phase II, participants were screened to determine study eligibility which consisted of the following criteria: (1) aged 18–31 years old; (2) mentally and physically healthy; (3) no contraindication to MRI (e.g., no pacemaker or metal in the body), and (4) registered with a general practitioner in the UK. Ethical approval was obtained from King's College London Research Ethics Committee and participants gave written informed consent to take part in the study. In addition, a subset of participants provided consent for their demographic and defaced neuroimaging data to be made publicly available to other researchers at the end of the study.

### 2.2 Experimental design

At Phase I and II, participants attended King's College London for two visits approximately three weeks apart for an MRI scan.

At Phase I, all scans were acquired on Scanner 1 (details below). Participants first received two identical MRI scans (A1 & A2). Participants were then asked to get out and immediately re-enter the scanner (A3), which intended to replicate longitudinal study designs where each scan might be associated with persons’ positioning within the scanner. For the last scan, participants were instructed to tilt their head down by approximately ten degrees (A4); *see* Supplementary S1 eFig. 1 for graph showing mean head tilt (pitch) movement of 7.61°. This movement in pitch, and the resulting change in vertical translation of the head, has been reported as the most common head movement of adults during an MRI scan (Cusack et al., 2017). Localizer scans were administered prior to the A1 and A3 scans. This procedure was repeated at the second visit (B1 – B4).

**Fig. 1:**
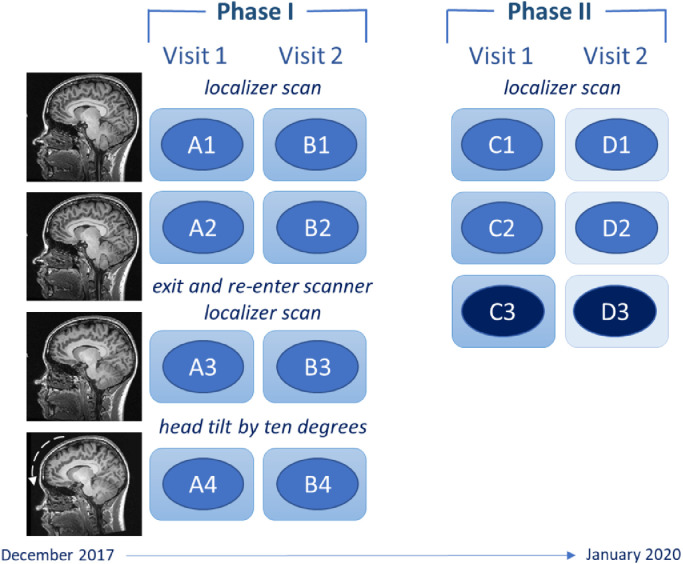
Illustration of the structural MRI data acquisition process in Phase I and II of the PIN study. In Phase II, the order of visits was balanced for participants so the D1 – D3 scans (on Scanner 2) were acquired first for ten participants. C3 and D3 scans were acquired using a different MRI sequence.

For Phase II visits, participants received three MRI scans on both Scanner 1 (C1 – C3) and Scanner 2 (D1 – D3). Scanners 1 and 2 were different scanners but the same model (details below). The order of scanner used first was manually balanced for the participants. This procedure aimed to examine inter-scanner variability. The third scan of each visit (C3 & D3) was acquired using a different MRI sequence compared to all other scans to explore inter-sequence variability (*see* Fig. 1 for data acquisition process).

### 2.3 MRI data acquisition

Structural MRI data were acquired using 3T MR750 Discovery GE (GE Healthcare, Chicago, USA) scanners: Scanner 1 (Software Version DV25.0_R02) and Scanner 2 (Software Version DV25.1_R02). MRI scanners did not undergo software updates during the course of the study and the GE standard distortion correction, “Gradwarp”, was enabled on the scanners.

The primary acquisition used was the T1 ADNI-3 sequence; an accelerated sagittal IR-FSPGR (Repetition Time (TR) = 7.36 ms, Echo Time (TE) = 3.05 ms, Inversion Time (TI) = 400 ms, Flip Angle (FA) = 11°, Voxel Size (VS) = 1 × 1 × 1 mm, Matrix Size = 256 × 256, Field of View (FOV) = 256, 196 slices). The ADNI-3 sequence was chosen as it has been specifically developed for longitudinal multi-centre studies where similar image qualities, such as reliability, contrast-to-noise, and spatial resolution, must be achieved across sites and over time (Jack Jr et al., 2008).

At Phase II, C3 and D3 scans were acquired using the ADNI-2 non-accelerated sagittal T1-weighted sequence (TR = 7.34 ms, TE = 3.04 ms, TI = 400 ms, FA = 11°, VS = 1 × 1 × 1.2 mm, Matrix Size = 256 × 256, FOV = 260, 196 slices). In addition to the ADNI consortium, the ADNI-2 sequence has previously been used in several international studies of mental health disorders [e.g., EU-GEI (Modinos et al., 2020) and PSYSCAN (Tognin et al., 2020)].

### 2.4 MRI data processing

To examine the effect of software version, FreeSurfer v5.3.0, v6.0.0 and v7.1.0 (Martinos Center for Biomedical Imaging) were used for structural MRI data pre-processing, segmentation and parcellation. The FreeSurfer automated stream consists of several processing steps, such as skull stripping, Talairach transformation, subcortical structure labelling, surface extraction, spherical registration and cortical parcellation. For each subject, we used the FreeSurfer cross-sectional and longitudinal streams. For cross-sectional analysis, the scans at every time point are processed independently for each subject. For longitudinal analysis, FreeSurfer uses results from the cross-sectional analysis of all time points to create an unbiased within-subject template; both of which are then used to initialise the final longitudinal processing stage (Reuter et al., 2010; Reuter et al., 2012). This three-stage procedure reduces variability and increases morphometry reproducibility compared to the cross-sectional analysis (Reuter et al., 2012). Scans were processed for the longitudinal stream in four groups: (1) Phase I (A1 – B4); (2) Phase I and II for nine participants (A1 – C2); (3) Phase II C1 and C3 data; and (4) Phase II ADNI-3 scans (C1, C2, D1 & D2).

The data was inspected for hard failures (i.e., where pre-processing is aborted) and screened for the presence of soft failures by manual visual inspection following the protocol of Iscan et al. (2015). No hard failures were seen, and no manual edits were made. Structural brain measurements were not impacted by normal variation in physiological variables, including the time of day of scans, blood pressure and hydration levels (Zahid et al., 2021).

For the reliability analysis, we analysed volume, surface area and thickness morphometry measurements generated from FreeSurfer's subcortical segmentation (ASEG) and cortical parcellation (APARC). We excluded some subcortical structures that were highly skewed, such as WM-hypointensities and 5th ventricle (cavum septum pellucidum). Further, we did not include the estimated total intracranial volume as this measure remains fixed for a given subject during the longitudinal processing stream (*see* Supplementary Materials S2 and S3 for brain regions included). In the Results Section 3.2.1, we determined that the head tilt condition was associated with reduced cortical thickness. To investigate this further, we used local values of the contrast-to-noise ratio and the number of surface holes (which indicate topological defects in the segmentation of the cortex); both of which are reported by FreeSurfer.

### 2.5 Statistical analyses

Reliability analysis of morphometric measurements was conducted in RStudio (RStudio Team, 2021) using the *irr* (Gamer et al., 2019) and *ggseg* (Mowinckel and Vidal-Piñeiro, 2020) packages. We calculated two-way mixed model intra-class correlation coefficients with absolute agreement of single measurements (McGraw and Wong, 1996):

$$\text{ICC}\;=\frac{MSr-MSe}{MSr+\left(k-1\right)MSe+\frac{k}{n}\left(MSc-MSe\right)\;}$$

where MSr is the mean square for rows (i.e., between-scan measurements), MSe is the mean square for error, MSc is the mean square for columns (i.e., within-scan measurements) and k is the number of measurements. Identical brain volumes produce an ICC value of 1.0. For volumetric studies, an ICC value greater than 0.9 indicates excellent reliability (Schnack et al., 2004).

Supplementary analyses were also carried out to calculate the mean absolute percentage differences (MPD) using the following equation:

$$\text{MPD}\;=\frac{\sum_{i=1}^{n}\left|\frac{\left(x_{j}-\;y_{j}\right)}{x_{j}}\right|}{n}\times \;100$$

where x is the reference scan, i.e., A1, y is the scan of interest, i.e., A2, and j represents the participant. Low MPD values indicate low variability and therefore, values closest to 0 are considered to be the most reproducible (McGuire et al., 2017). A random sample of ICCs and MPDs were validated in IBM SPSS Statistics 26 (IBM) to verify accuracy.

To compare the reliability of FreeSurfer processing, ICC and MPD values were calculated for data generated from both the cross-sectional and longitudinal streams of each FreeSurfer version. For each morphometric measure, seven reliability estimates were calculated. Structural T1-weighted ADNI-3 A1 data was compared to: (1) A2 (referred to as repetition), (2) A3 (reposition), (3) B1 (three-weeks), (4) C1 (one-year). In addition, A3 data was compared to A4 data (head tilt), C1 to C3 data (sequence change) and C1 to D1 data (scanner change) (*refer* back to Fig. 1). To reduce the number of analyses, we focused on comparisons that may have implications for longitudinal studies, therefore B2–B4, C2 and D2-D3 scans were not compared in the current paper.

Post-hoc statistical power calculations were carried out to determine the sample size required to detect longitudinal changes of predetermined magnitudes in regional thickness measurements, based on recent work by Laguna et al. (2020). Specifically, we calculated the minimum number of participants needed to detect 0.5%, 1.0% and 2.0% group-level changes from baseline to (a) three-weeks and (b) after head tilt. These percentage changes are in line with those reported in examples of neurological and psychiatric disorders (Olabi et al., 2011; Frings et al., 2014). To note, the selected values relate to 0.5%, 1.0% and 2.0% changes in FreeSurfer measurement and may not directly correspond to the same biological change. For the power calculations, we obtained the effect size relating to a paired *t*-test on the difference between two dependant means (Cohen, 1988). We used the *in-vivo* MRI data for (a) A1 and B1 scans for three weeks and (b) A3 and A4 scans for head tilt to estimate the population means (μ_x_ and μ_y_), population variances (σ_x_^2^ and σ_y_^2^) and the correlation between the observations (ρ_xy_). To calculate changes of a predetermined magnitude, we replaced | μ_x_ - μ_y_ | with | μ_x_ * *pc* | (for more details *see* Laguna et al., 2020) so that:

$$d_{z}=\frac{\left|\;\mu_{x}\;*\;pc\;\right|\;}{\sqrt{\sigma_{x}^{2}+\;\sigma_{y}^{2}\;-\;2\rho_{xy}\sigma_{x}^{2}\sigma_{y}^{2}}}$$

where *pc* = 0.005, 0.01, 0.02. In G*Power (Faul et al., 2007), we entered the computed effect size, *d*_z_, statistical significance level α = 0.05 (two-tailed) and statistical power of at least 1 − β = 0.8.

## 3 Results

### 3.1 Participants

Twenty healthy participants (11 female and 9 male, aged = 20–30, mean age = 24.0 ± 2.9) took part in Phase I of the study. Mean interval between participants’ first and second visit of Phase I was 20.3 ± 16.1 days. Nine Phase I participants and 11 new participants completed Phase II (11 female and 9 male, aged = 22–31, mean age = 24.7 ± 2.5). For the nine returning participants, the mean number of days from the start of Phase I to Phase II was 474.7 ± 43.2. For Phase II, mean interval between 20 participants’ first and second visit was 9.1 ± 4.0 days. Demographic and structural MRI data for a subset of 24 participants is publicly available to download at https://sites.google.com/view/pinstudy.

### 3.2 Morphometric measure reliability

#### 3.2.1 Impact of image acquisition factors

Table 1 shows mean ICC values of subcortical and cortical volumes, cortical thickness, and cortical surface area for the seven planned comparisons using FreeSurfer v7.1.0 longitudinal stream (*see* Supplementary Materials S2 for ICC values of individual brain regions). There were high values of reliability for all subcortical and cortical volumes and cortical surface area comparisons, with values ranging from 0.964 – 0.990 (95% confidence intervals (CI): 0.804 – 0.997). Cortical thickness appears to be the least reliable morphometric measure with an ICC range of 0.736 – 0.926 (95% CI: 0.352 – 0.970). As a result, using data presented in Supplementary Materials S2, we calculated the mean ICC (across the seven planned comparisons) for thickness estimates of individual regions. Five regions with highest cortical thickness reliability were the right hemisphere caudal anterior cingulate, precentral gyrus, isthmus cingulate, and right and left hemisphere parahippocampus (ICCs of 0.937 – 0.969). Those with the lowest reliability estimates were the right hemisphere supramarginal gyrus, superior parietal lobe, temporal pole, and right and left hemisphere inferior parietal lobes (ICCs of 0.714 – 0.754).

**Table 1:** Mean ICC values of subcortical and cortical volumes, cortical thickness, and cortical surface area morphometric measurements from FreeSurfer v7.1.0 longitudinal stream.

LH, left hemisphere; RH, right hemisphere.

^1^ generated from subcortical segmentation (aseg.stats) of FreeSurfer v7.1.0 longitudinal stream.

^2^ generated from cortical parcellation (aparc.stats) of FreeSurfer v7.1.0 longitudinal stream.

^3^ calculated for each comparison as the mean ICC value of subcortical volume and cortical volume, thickness, and surface area measurements.

^4^*n* = 9 participants.

**Fig. 2:**
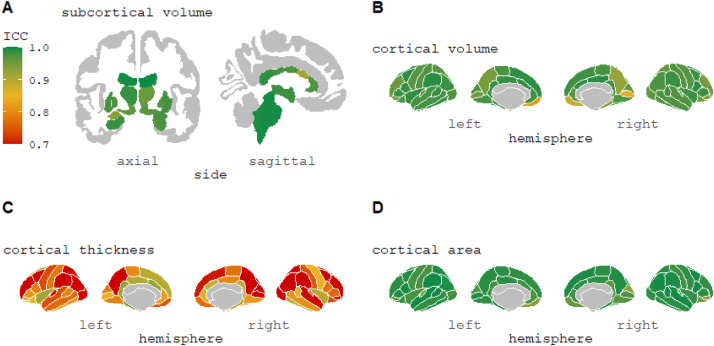
ICC values of subcortical and cortical volumes, cortical thickness, and cortical surface area morphometric measurements for ‘head tilt’ comparison from FreeSurfer 7.1.0 longitudinal stream. This change in pitch has been reported as the most common type of head movement seen in adult groups during an MRI scan (Cusack et al., 2017). For cortical thickness measurements (Image C), 16 regions had an ICC<0.7 (minimum value = 0.402) (see Supplementary Materials S2).

**Fig. 3:**
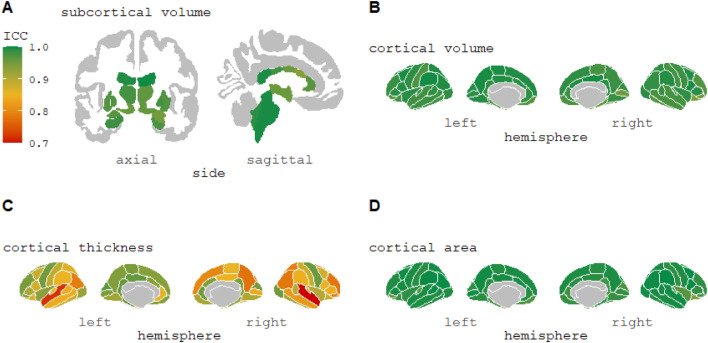
ICC values of subcortical and cortical volumes, cortical thickness, and cortical surface area morphometric measurements for ‘three-week’ comparison from FreeSurfer 7.1.0 longitudinal stream. For cortical thickness measurements (Image C), two regions had an ICC<0.7 (minimum value = 0.598) (see Supplementary Materials S2).

From Table 1, asking participants to tilt their head in the scanner (mean pitch movement of 7.61°) resulted in lower reliability of morphometric measurements (overall mean ICC = 0.909) compared to follow-up scans at three weeks (ICC = 0.951) or one year (ICC = 0.940). The reliability of individual brain regions is shown for ‘head tilt’ (Fig. 2) and ‘three weeks’ (Fig. 3).

**Fig. 4:**
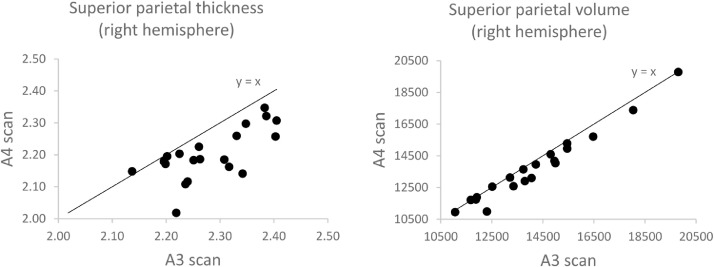
Left: scatterplot of right superior parietal **thickness** (mm) showing the effect of head tilt (A4 scan), which is associated with relatively low reliability (ICC=0.40) and an apparent reduction in cortical thickness. Right: scatterplot for **volume** (mm^3^) comparison of the same region is shown where there is higher reliability (ICC=0.96).

To understand the low reliability of cortical thickness measurements after the head tilt, seen in Fig. 2C, we first plotted the thickness estimates from A3 and A4 scans for a cortical region which was particularly impacted by head movement. Fig. 4 indicates that head tilt movement is associated with an approximate 3 – 4% decrease in cortical thickness measurement. Using a paired samples *t*-test, we further observed a significant global reduction in mean cortical thickness following the head tilt (t[19] = −6.17, *p* <0.001). Second, we tested whether the cortical thinning was associated with the difference in contrast-to-noise ratio or the number of surface holes after the head tilt amongst five cortical regions with the lowest reliability (listed in Section 3.2.1 above). The contrast-to-noise ratio was significantly reduced in the head tilt condition for four of the cortical regions (all *p* <0.01). Results indicated that lower contrast-to-noise ratio after the head tilt was associated with a reduction in cortical thickness for all five regions (Pearson's *r* of 0.46 to 0.62, all *p* <0.05). In contrast, no significant correlations were reported for the difference in cortical thickness and surface hole measures before and after the head tilt (all *p* >0.05).

**Fig. 5:**
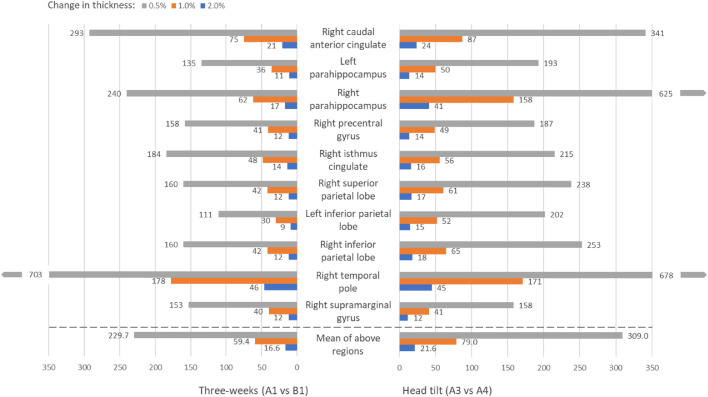
Statistical power of ‘three-week’ and ‘head tilt’ comparisons across ten regional thicknesses of the highest and lowest reliability. Graph displays the sample size needed to detect longitudinal changes in thickness at *p*<0.05 significance level and 1-β statistical power = 0.80 (two-tailed). For example, for a study to be sufficiently powered to detect a 1% longitudinal change in the left parahippocampal thickness, 36 participants would be required using data from the three-week scan. However, 50 participants would be needed if there was a change in head tilt.

Post-hoc statistical power analyses were conducted to determine the minimum number of participants needed to detect 0.5%, 1.0% and 2.0% longitudinal changes in cortical thickness from baseline to (a) three-week scan compared to (b) scan after the head tilt. For a wide range of reliability, the five cortical regions showing the highest and lowest reliability in Section 3.2.1 were used. Fig. 5 shows that a larger number of participants are typically required to detect these changes as a result of a head tilt after the localizer scan.

#### 3.2.2 Impact of image processing factors

Table 2 shows mean ICC values of subcortical and cortical volumes, cortical thickness, and cortical surface area for the seven planned comparisons using FreeSurfer v7.1.0 *cross-sectional* stream. Mean ICCs ranged from 0.706 – 0.950 (*see* Supplementary Materials S2 for ICC values of individual brain regions).

**Table 2:** Mean ICC values of subcortical and cortical volumes, cortical thickness, and cortical surface area morphometric measurements from FreeSurfer v7.1.0 cross-sectional stream.

LH, left hemisphere; RH, right hemisphere.

^1^ generated from subcortical segmentation (aseg.stats) of FreeSurfer v7.1.0 cross-sectional stream.

^2^ generated from cortical parcellation (aparc.stats) of FreeSurfer v7.1.0 cross-sectional stream.

^3^ calculated for each comparison as the mean ICC value of subcortical volume and cortical volume, thickness, and surface area measurements.

^4^*n* = 9 participants.

From Table 3, we can begin to compare the reliability of the different FreeSurfer processing streams using data from the ‘three weeks’ comparison. FreeSurfer v7.1.0, v6.0.0 and v5.3.0 longitudinal streams displayed high reliability across morphometry measurements (overall FreeSurfer mean ICCs of 0.930 – 0.951) and demonstrated greater reliability than the cross-sectional streams (overall FreeSurfer mean ICCs of 0.829 – 0.887).

**Table 3:** Mean ICC values of subcortical and cortical volume, cortical thickness, and cortical surface area morphometric measurements from three-weeks (A1 vs B1) comparison.

LH, left hemisphere; RH, right hemisphere.

^1^ generated from subcortical segmentation (aseg.stats).

^2^ generated from cortical parcellation (aparc.stats).

^3^ calculated for each FreeSurfer processing stream as the mean ICC value of subcortical volume and cortical volume, thickness, and surface area measurements.

### 3.3 Supplementary analyses using mean absolute percentage difference

Focusing on results from the FreeSurfer v7.1.0 longitudinal stream, the mean absolute percentage difference (MPD) values for cortical volume, surface area and thickness measurements supported the ICC results, where head tilt had the greatest adverse effect on reproducibility. Generally, the MPD values were higher for subcortical volume measurements (1.948% to 2.852%), which were particularly impacted by poor inferior lateral ventricle reproducibility, and were more affected by the inter-scan interval, and change in MRI sequence or scanner (Supplementary S1 eTable 1). From the MPD results, we observed that brain measurements processed by FreeSurfer v7.1.0 longitudinal stream typically had higher reproducibility (mean MPD of 1.978%) than the cross-sectional stream and earlier FreeSurfer versions (mean MPDs of 2.046% to 5.038%) (Supplementary S1 eTables 2 and 3). Results for individual brain regions have been provided in Supplementary Materials S3.

## 4 Discussion

We reported a minimal decrease in reliability of longitudinal MRI measures from within-session to three weeks and one year. Generally, head tilt had a greater adverse impact on reliability of brain measurements compared to participant repositioning and changes in MRI sequence or scanner. Notably, the study demonstrated the reliability advantages of the FreeSurfer longitudinal stream compared to the cross-sectional stream. These findings were supported by supplementary MPD analyses. To our knowledge, this is the first study to provide ICC reliability and MPD values for all FreeSurfer-segmented regions, which may inform power calculations and study design for future longitudinal MRI studies.

We first demonstrated how the test-retest reliability of MRI-derived measurements are impacted by image acquisition factors, namely scan session (repetition), inter-scan interval (three weeks and one year), participant positioning, head tilt, MRI sequence and scanner. Focusing on results obtained from the FreeSurfer v7.1.0 longitudinal stream, repetition had the greatest reliability but the ICCs at three weeks and one year still indicated high reliability of structural brain measurements. These results are in line with early findings by Jovicich et al. (2009) who reported comparable within- and between-session reliability of subcortical volume estimates from FreeSurfer v4.0. In our study, the small decrease in reproducibility of brain measurements at one year may reflect physiological changes, such as normal ageing (Scahill et al., 2003). In general, the reliability of brain measurements, and particularly cortical thickness, seemed to be most affected by participants’ head tilt. Furthermore, post-hoc statistical power calculations showed that, on average, an increased number of participants would be needed to detect longitudinal thickness changes following a head tilt compared to three-week scan. We have demonstrated evidence that reduced cortical thickness in the head tilt condition may arise due to reduced contrast-to-noise ratio in the MRI data. Although other head rotations could also have had an impact, we chose to examine the change in pitch (i.e., chin moving towards the neck) as this is a common motion over time during long scans. The head tilt (of seven degrees) occurred after the localizer image, but before the start of the scan, which may occur in a clinical study if the structural scan is acquired later in the protocol and the participant's head has since moved. Previous research has only reported on the effects of head motion during a scan, which can also lead to systematic biases in brain estimation (Alexander‐Bloch et al., 2016; Reuter et al., 2015; Savalia et al., 2017). As well as reducing head motion during the scan (Reuter et al., 2015), we recommend that studies monitor or control for head tilt after the localizer scan. Furthermore, we reported that comparisons of different MRI sequences and scanners produced high reliability of brain measurements. Similarly, Jovicich et al. (2009) have shown comparable reliability results of two imaging sequences (MPRAGE and multi-echo FLASH), indicating that FreeSurfer segmentation of brain volumes is robust across similar image contrast properties. In a more recent study of 24 participants scanned across three models of Siemens MRI scanners, Sederevičius et al. (2021) reported high test-retest reliability of FreeSurfer v7.1.0 automated subcortical segmentation (ASEG). However, ASEG showed increased inter-scanner variability of repeated measures compared to Sequence Adaptive Multimodal Segmentation approach, newly released as part of FreeSurfer v7.1 software. As the ICC values for both the MRI sequence and scanner comparisons are similar to those at three weeks (using the same sequence and scanner), this suggests that these sequences and GE MR750 scanner could be combined in a longitudinal MRI study.

The present study examined the test-retest reliability of subcortical and cortical volume, cortical surface area, and cortical thickness estimates. For FreeSurfer v7.1.0 longitudinal processing, we found that volume and surface area measurements appeared to be more reliable measures, whereas cortical thickness estimates had lower reliability. Specifically, our findings indicated high reproducibility of cortical thickness measurements for scans obtained within the same session (repetition and subject repositioning), but reliability estimates did vary by brain region and were more affected by changes in acquisition and processing factors, such as head tilt, one year scan and MRI scanner or sequence change. This finding is similar to previous studies (Han et al., 2006; McGuire et al., 2017). Authors reported that cortical thickness measurements showed excellent ICC values but were impacted by imaging sequence and field strength (Han et al., 2006) and were lower for certain brain structures, including the entorhinal, insula and medial orbitofrontal (McGuire et al., 2017). For particular brain regions with low ICC reliability, it may be beneficial to use other software or even manual tracing measures to increase precision (Du et al., 2020). Overall, we determined that FreeSurfer-derived measurements are highly reproducible, particularly when acquisition and processing factors are controlled for. Therefore, it is important to consider these factors during the design of a longitudinal MRI study (Han et al., 2006).

Looking at the effect of image processing factors on reliability, our study highlighted the advantages of using the FreeSurfer v7.1.0 longitudinal stream. In line with previous findings (Han et al., 2006; Jovicich et al., 2013), our results showed that the longitudinal stream offers an improvement of reliability for both within- and between-session brain measurements compared to the cross-sectional stream. Furthermore, our results demonstrated increasing reliability of software versions over time (from v5.3.0 to v7.1.0), although the longitudinal streams of v7.1.0 and v6.0.0 showed similar reliability. Earlier reliability studies have also reported an effect of FreeSurfer software version (from v4.1.0 to v5.1.0) on cortical thickness estimates (Chepkoech et al., 2016; Gronenschild et al., 2012), suggesting that reliability is affected by FreeSurfer processing conditions. Due to version differences, we recommend that consistent FreeSurfer versions should be used for MRI data processing in ongoing longitudinal studies.

The present study has several strengths and limitations. First, we have conducted and shared a comprehensive reliability assessment of structural brain measurements in a group of healthy participants. Further, we have made the raw, defaced MRI data for a subset of participants publicly available to other researchers (https://sites.google.com/view/pinstudy). A third strength is that we selected the widely used ADNI-3 sequence for MRI acquisition which has been specifically developed for multi-site studies (Jack Jr et al., 2008) and is likely to be used in future longitudinal MRI studies. A limitation is that our sample consisted of 20 healthy individuals, which limits the interpretation and generalisability of our results (i.e., to samples of older adults and individuals with neurodegenerative disorders). However, this study recruited young adults as our clinical research focuses on mental health disorders, where the typical age of onset is in late adolescence and early adulthood (Kessler et al., 2008) and where we expect to see much smaller structural brain changes over time (Olabi et al., 2011). In addition, our sample size of healthy participants is similar to, if not greater than, previous reliability studies (Du et al., 2020; Jovicich et al., 2009; McGuire et al., 2017; Morey et al., 2010; Reuter et al., 2015). Furthermore, longitudinal neuroimaging studies require smaller sample sizes to detect small effect size differences in brain structure compared to cross-sectional studies (Steen et al., 2007). For example, for brain regions with ICCs close to 1.0, a longitudinal study requires approximately ten participants to be powered to detect small to large effect sizes (Morey et al., 2010). Another limitation is that, although we obtained data from two MRI scanners, they were both GE Discovery MR750 scanners. Therefore, our results cannot necessarily be generalised to MRI data combined from different scanner manufacturers in multi-centre studies.

As accurate and reliable automated segmentation is essential for longitudinal studies (Sederevičius et al., 2021), it is crucial to examine and report the reproducibility of quantitative brain morphometry results (Jovicich et al., 2006). Overall, the present study demonstrated high test-retest reliability of FreeSurfer-derived measurements and showed superiority of FreeSurfer v7.1.0 and v6.0.0 longitudinal streams. However, reliability of measurements still varied depending upon other image acquisition factors. Cortical thickness reliability estimates were lower for certain brain regions and so researchers may want to consider using alternative software, manual tracing methods, or even excluding these regions in their analysis. As head tilt seemed to have the greatest impact on reliability of structural brain measurements, we suggest that future longitudinal studies monitor and/or control head tilt after the localizer scan.

## Data availability statement

Data is available from https://sites.google.com/view/pinstudy for a subset of participants who provided consent for their demographic and defaced neuroimaging data to be made publicly available.

## CRediT authorship contribution statement

**Emily P Hedges:** Methodology, Software, Validation, Formal analysis, Investigation, Data curation, Writing – original draft, Writing – review & editing, Visualization. **Mihail Dimitrov:** Methodology, Software, Investigation, Data curation, Writing – review & editing. **Uzma Zahid:** Methodology, Investigation, Data curation, Writing – review & editing. **Barbara Brito Vega:** Investigation, Writing – review & editing. **Shuqing Si:** Validation, Formal analysis, Writing – review & editing, Visualization. **Hannah Dickson:** Writing – review & editing. **Philip McGuire:** Writing – review & editing. **Steven Williams:** Writing – review & editing, Funding acquisition. **Gareth J Barker:** Methodology, Writing – review & editing. **Matthew J Kempton:** Conceptualization, Methodology, Resources, Writing – review & editing, Visualization, Supervision, Project administration, Funding acquisition.

## Declaration of Competing Interest

The authors declare no conflict of interest.

## Appendix Supplementary materials

Image, application 1

Image, application 2

Image, application 3
