# Supplementary material for: Reliability of structural MRI measurements: The effects of scan session, head tilt, inter-scan interval, acquisition sequence, FreeSurfer version and processing stream
Source: Neuroimage. 2022 Feb 1;246:118751. doi: 10.1016/j.neuroimage.2021.118751 (PMC8784825; doi:10.1016/j.neuroimage.2021.118751)
Supplement: Supplementary file 1 [file mmc1.docx]

**Supplementary Materials S1**

**eFigure 1.** Graph demonstrating head tilt forward (pitch) of 7.61^o^ in A3 vs A4 compared to the other comparisons in Phase I

**eTable 1.** Mean absolute percentage difference (MPD) values of subcortical and cortical volumes, cortical thickness, and cortical surface area morphometric measurements from FreeSurfer v7.1.0 longitudinal stream

**eTable 2.** MPD values of subcortical and cortical volumes, cortical thickness, and cortical surface area morphometric measurements from FreeSurfer v7.1.0 cross-sectional stream

**eTable 3.** MPD values of subcortical and cortical volumes, cortical thickness, and cortical surface area morphometric measurements from three-weeks (A1 vs B1) comparison

**eFigure 1:** Graph demonstrating head tilt forward (pitch) of 7.61^o^ in A3 vs A4 compared to the other comparisons in Phase I. A movement in pitch indicates a forward head movement (i.e., moving chin towards the neck, or nodding the head), in comparison to roll and yaw which refer to a tilting movement (i.e., tilting head to one shoulder) and a horizontal movement (i.e., shaking the head), respectively. Error bars are the standard error of the mean (SEM).

**eTable 1.** Mean absolute percentage difference (MPD) values of subcortical and cortical volumes, cortical thickness, and cortical surface area morphometric measurements from FreeSurfer v7.1.0 longitudinal stream

LH, left hemisphere; RH, right hemisphere

^1^generated from subcortical segmentation (aseg.stats) of FreeSurfer v7.1.0 longitudinal stream

^2^generated from cortical parcellation (aparc.stats) of FreeSurfer v7.1.0 longitudinal stream

^3^calculated for each comparison as the mean MPD value of subcortical volume and cortical volume, thickness, and surface area measurements

^4^n=9 participants

|  |  | Repetition A1 vs A2 | Reposition A1 vs A3 | Head tilt A3 vs A4 | Three weeks A1 vs B1 | One year^4^ A1 vs C1 | Sequence C1 vs C3 | Scanner C1 vs D1 |
| --- | --- | --- | --- | --- | --- | --- | --- | --- |
| Subcortical volume^1^ | | 1.948 | 2.369 | 2.512 | 2.781 | 2.653 | 2.852 | 2.692 |
| Cortical volume^2^ | LH | 1.551 | 1.572 | 2.673 | 1.925 | 2.261 | 2.511 | 2.093 |
|  | RH | 1.670 | 2.006 | 2.651 | 2.171 | 2.542 | 2.248 | 1.976 |
| Cortical thickness^2^ | LH | 1.444 | 1.459 | 2.561 | 1.721 | 1.874 | 2.029 | 1.848 |
|  | RH | 1.751 | 1.860 | 2.644 | 2.035 | 2.013 | 1.947 | 1.935 |
| Cortical surface area^2^ | LH | 1.257 | 1.491 | 1.930 | 1.579 | 1.700 | 1.697 | 1.718 |
|  | RH | 1.478 | 1.651 | 2.000 | 1.635 | 1.706 | 1.850 | 1.561 |
| Comparison mean value^3^ | | 1.585 | 1.772 | 2.425 | 1.978 | 2.107 | 2.162 | 1.975 |

**eTable 2.** MPD values of subcortical and cortical volumes, cortical thickness, and cortical surface area morphometric measurements from FreeSurfer v7.1.0 cross-sectional stream

LH, left hemisphere; RH, right hemisphere

^1^generated from subcortical segmentation (aseg.stats) of FreeSurfer v7.1.0 cross-sectional stream

^2^generated from cortical parcellation (aparc.stats) of FreeSurfer v7.1.0 cross-sectional stream

^3^calculated for each comparison as the mean MPD value of subcortical volume and cortical volume, thickness, and surface area measurements

^4^n=9 participants

|  |  | Repetition A1 vs A2 | Reposition A1 vs A3 | Head tilt A3 vs A4 | Three weeks A1 vs B1 | One year^4^ A1 vs C1 | Sequence C1 vs C3 | Scanner C1 vs D1 |
| --- | --- | --- | --- | --- | --- | --- | --- | --- |
| Subcortical volume^1^ | | 3.535 | 3.817 | 3.931 | 4.198 | 4.339 | 5.238 | 3.741 |
| Cortical volume^2^ | LH | 4.062 | 4.015 | 4.967 | 4.163 | 4.532 | 6.060 | 3.990 |
|  | RH | 3.718 | 4.109 | 4.774 | 4.025 | 5.296 | 6.816 | 4.444 |
| Cortical thickness^2^ | LH | 2.425 | 2.364 | 3.117 | 2.654 | 2.816 | 3.475 | 2.490 |
|  | RH | 2.327 | 2.501 | 3.219 | 2.829 | 2.915 | 3.578 | 2.685 |
| Cortical surface area^2^ | LH | 3.528 | 3.874 | 4.477 | 3.454 | 3.590 | 4.586 | 3.830 |
|  | RH | 3.374 | 3.824 | 4.255 | 3.586 | 4.491 | 5.135 | 3.974 |
| Comparison mean value^3^ | | 3.281 | 3.501 | 4.106 | 3.558 | 3.997 | 4.984 | 3.594 |

**eTable 3.** MPD values of subcortical and cortical volumes, cortical thickness, and cortical surface area morphometric measurements from three-weeks (A1 vs B1) comparison

LH, left hemisphere; RH, right hemisphere

^1^generated from subcortical segmentation (aseg.stats)

^2^generated from cortical parcellation (aparc.stats)

^3^calculated for each FreeSurfer processing stream as the mean MPD value of subcortical volume and cortical volume, thickness, and surface area measurements

|  |  | FreeSurfer longitudinal stream | | | FreeSurfer cross-sectional stream | | |
| --- | --- | --- | --- | --- | --- | --- | --- |
|  |  | 7.1.0 | 6.0.0 | 5.3.0 | 7.1.0 | 6.0.0 | 5.3.0 |
| Subcortical volume^1^ | | 2.781 | 2.774 | 3.094 | 4.198 | 3.849 | 5.831 |
| Cortical volume^2^ | LH | 1.925 | 2.134 | 2.823 | 4.163 | 4.447 | 5.323 |
|  | RH | 2.171 | 2.307 | 2.940 | 4.025 | 4.676 | 6.474 |
| Cortical thickness^2^ | LH | 1.721 | 1.712 | 2.412 | 2.654 | 2.735 | 3.535 |
|  | RH | 2.035 | 2.059 | 2.816 | 2.829 | 2.849 | 3.893 |
| Cortical surface area^2^ | LH | 1.579 | 1.606 | 1.763 | 3.454 | 4.041 | 4.763 |
|  | RH | 1.635 | 1.732 | 1.780 | 3.586 | 4.168 | 5.449 |
| FreeSurfer mean value^3^ | | 1.978 | 2.046 | 2.518 | 3.558 | 3.824 | 5.038 |
